# Chinese Nian Gao Inspired Textured Janus Hydrogel for Body Signal Sensing and Human Machine Interaction

**DOI:** 10.1002/advs.202509573

**Published:** 2025-07-30

**Authors:** Haiyu Li, Hui Zhang, Xinxin Liu, Jing Jie, Ming Yin, Jie Du

**Affiliations:** ^1^ School of Materials Science and Engineering Hainan University Haikou 570228 China; ^2^ Shandong Key Laboratory of Preparation and Application of New Thermoplastic Elastomer Materials Shandong 265702 China; ^3^ State Key Laboratory of Digital Medical Engineering Key Laboratory of Biomedical Engineering of Hainan Province School of Biomedical Engineering Hainan University Sanya Hainan 572025 China

**Keywords:** assistive robotic arm, asymmetric janus adhesion, electromyographic (EMG) and electroencephalogram (EEG) signals, health monitoring, human–machine interaction, multifunctional hydrogel

## Abstract

Inspired by Chinese Nian Gao, a traditional food in China, a skin‐like bio‐hydrogel with asymmetric Janus adhesion and textured structures on its surface is fabricated via a one‐pot strategy and is evaluated as electronic skin for sensing and human–machine interaction. The e‐skin is constructed through hydrogen bonding and metal‐ligand coordination with favorable toughness and stretchability (tensile strength of 173 kPa, strain of 1593%). The bottom surface of the e‐skin reached an adhesion strength of 66.7 kPa, and the upper surface shows no adhesion on different substrates, which ensures the stable signal collection on the bottom and avoids interference from incidental contact with clothing or external objects on the upper side. The skin‐mimic textures are created in drying, which endows the hydrogel with visual authenticity comparable to biological skin; consequently, it can be used for human scar coverage and skin encapsulation of humanoid robots. The e‐skin has an outstanding biocompatibility as well as a high self‐healing efficiency of 93.5% and it is developed and demonstrated for multipurpose real‐time applications. Furthermore, it accurately identifies hand gestures to control an assistive robotic arm in real‐time based on EMG and EEG signals, highlighting its potential in next‐generation aesthetic and functional wearable electronics.

## Graphical abstract

An e‐skin with asymmetric adhesion and textured surface is fabricated. It features an adhesive base and a nonadhesive upper interface, which enables efficient real‐time monitoring of human signals and ensures stable acquisition and transmission of EEG/EMG to facilitate robotic arm manipulation. The biomimetic surface texture combines aesthetic appeal with functional adaptability, demonstrating potential for epidermal integration and humanoid robotics encapsulation.

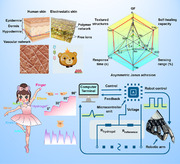

## 1 Introduction

Recent advancements in flexible electronics have propelled flexible sensors into the forefront of next‐generation sensing technologies, offering transformative solutions beyond the limitations of conventional rigid sensors.^[^ ^1^, ^2^, ^3^ ^]^ Unlike their brittle counterparts, flexible sensors exhibit exceptional conformability, stretchability, and biocompatibility, enabling seamless integration with dynamic biological surfaces and irregular geometries.^[^ ^4^, ^5^, ^6^, ^7^, ^8^, ^9^, ^10^ ^]^ These unique attributes make them indispensable for emerging applications ranging from personalized healthcare monitoring to adaptive human–machine interfaces.^[^ ^11^, ^12^, ^13^ ^]^ The rapid development of functional materials has expanded the toolkit for fabricating flexible sensors, such as spanning metal nanomaterials, conductive polymers, carbon‐based architectures, and liquid metals.^[^ ^14^, ^15^, ^16^, ^17^ ^]^ Among these, hydrogels have emerged as a particularly promising candidate for constructing high‐performance flexible sensors.^[^ ^18^, ^19^ ^]^ Characterized by their tissue‐like softness, tunable mechanical properties, and intrinsic biocompatibility, hydrogel‐based sensors mimic the compliance of biological systems while enabling sensitive detection of mechanical, thermal, and biochemical stimuli.^[^ ^20^, ^21^, ^22^, ^23^, ^24^, ^25^, ^26^ ^]^ When functionalized with conductive networks, these water‐rich polymers demonstrate exceptional strain sensitivity, fast response, and long‐term stability, surpassing many conventional flexible materials in human‐motion detection accuracy.^[^ ^27^, ^28^, ^29^, ^30^ ^]^

The unique features of hydrogel sensors position them as ideal platforms for developing multifunctional electronic skins (e‐skins).^[^ ^31^, ^32^ ^]^ A critical prerequisite lies in achieving robust interfacial adhesion between hydrogels and biological surfaces to ensure stable signal acquisition and conduction.^[^ ^33^ ^]^ Recent advances have yielded bio‐adhesive hydrogels through strategies like dopamine‐based catechol coupling, hydrogen‐bond reinforced networks, and dynamic Schiff‐base reactions.^[^ ^34^, ^35^, ^36^, ^37^ ^]^ However, emerging applications in soft robotics and human–machine interfaces demand more sophisticated adhesion control.^[^ ^38^ ^]^ Specifically, the bottom layer of an e‐skin should adhere firmly to signal sources such as skin or organ surfaces to achieve stable signal collection, while the upper surface should be non‐adhesive to avoid interference from incidental contact with clothing or external objects. Current approaches to unilateral adhesion rely on asymmetric surface modifications, such as oxygen‐inhibited polymerization for gradient crosslinking or plasma‐treated hydrophobic upper layers.^[^ ^39^, ^40^, ^41^ ^]^ While these methods achieve moderate adhesion contrast, they suffer from delamination risks under prolonged mechanical stress and require complex multi‐step fabrication.^[^ ^42^ ^]^

The construction of differential adhesion can indeed be achieved through simple inspirations that are readily observable in daily life. A notable example is Chinese sticky rice cake (so‐called Nian Gao in Chinese), a traditional delicacy consumed during Chinese New Year celebrations. Freshly steamed rice cake exhibits uniform strong adhesion across its entire surface. However, during storage, the upper surface undergoes dehydration‐induced densification of the hydrogen bond network. This structural reorganization enables molecular chains to form more compact configurations through enhanced hydrogen bonding, consequently reducing surface free energy and burying adhesive functional groups.^[^ ^43^ ^]^ In contrast, the bottom surface maintains strong adhesion due to its constrained contact with the plate, which prevents similar dehydration and preserves the original hydrogen bond network configuration. Such inherent Janus adhesion eliminates postprocessing steps and ensures operational stability.

Furthermore, controlled drying condition induces the formation of surface wrinkles on rice cakes, creating morphological features that bear resemblance to human skin textures. Such engineered skin‐like textures hold significant application potential in electronic skin technologies. For instance, they could be employed as conformal coatings on human body surfaces to minimize visual discrepancies with natural skin while maintaining sensing functionality, thereby achieving both aesthetic integration and functional performance.^[^ ^44^ ^]^ These textured structures also serve as promising candidates for humanoid robot epidermis, enhancing the biomimetic authenticity of robotic surfaces. Additionally, texture patterns mimicking fingerprint characteristics could improve signal transduction fidelity and feedback responsiveness in robotic tactile sensors, particularly during contact‐based sensing or precision manipulation tasks.^[^ ^45^, ^46^ ^]^ These advances collectively contribute to the development of bio‐inspired flexible sensing, human–machine interfaces, and perceptual enhancement in anthropomorphic robotics. Typically, the natural macromolecular chains in Nian Gao with high flexibility tend to form flat and dense layers rather than pronounced wrinkles. Nevertheless, this phenomenon offers valuable inspiration for texture engineering as the textured structure can be promoted by incorporating rigid structural components to amplify local stress inhomogeneity.

Herein, inspired by Chinese Nian Gao, an asymmetric Janus bio‐hydrogel with a biomimetic skin texture structure was prepared via a one‐pot strategy. The hydrogel was constructed based on hydrogen bonding and metal‐ligand coordination. The strong intermolecular interactions and adhesive groups promoted the self‐condensation on the upper surface, resulting in an asymmetric Janus adhering property. The hydrogen bonds and rigid framework provided by cellulose nanocrystals (CNC) combined with coordination bonds formed by Al^3+^ not only contribute to the asymmetry surface but also alter the stress distribution within the matrix, which offers robust support for the construction of surface textures. The textured Janus hydrogel was applied as e‐skin for body signal monitoring, human–machine interaction, and manipulation of the assistive robotic arm, and related properties such as mechanical strength, adhesive ability, self‐healing ability, and biocompatibility were evaluated. With its biomimetic functionality and detection stability, it shows great potential for next‐generation sensing technologies in intelligent robotic systems.

## 2 Results and Discussion

### 2.1 Design and Preparation of PPAlC_x_G_y_ Hydrogel

**Figure 1:**
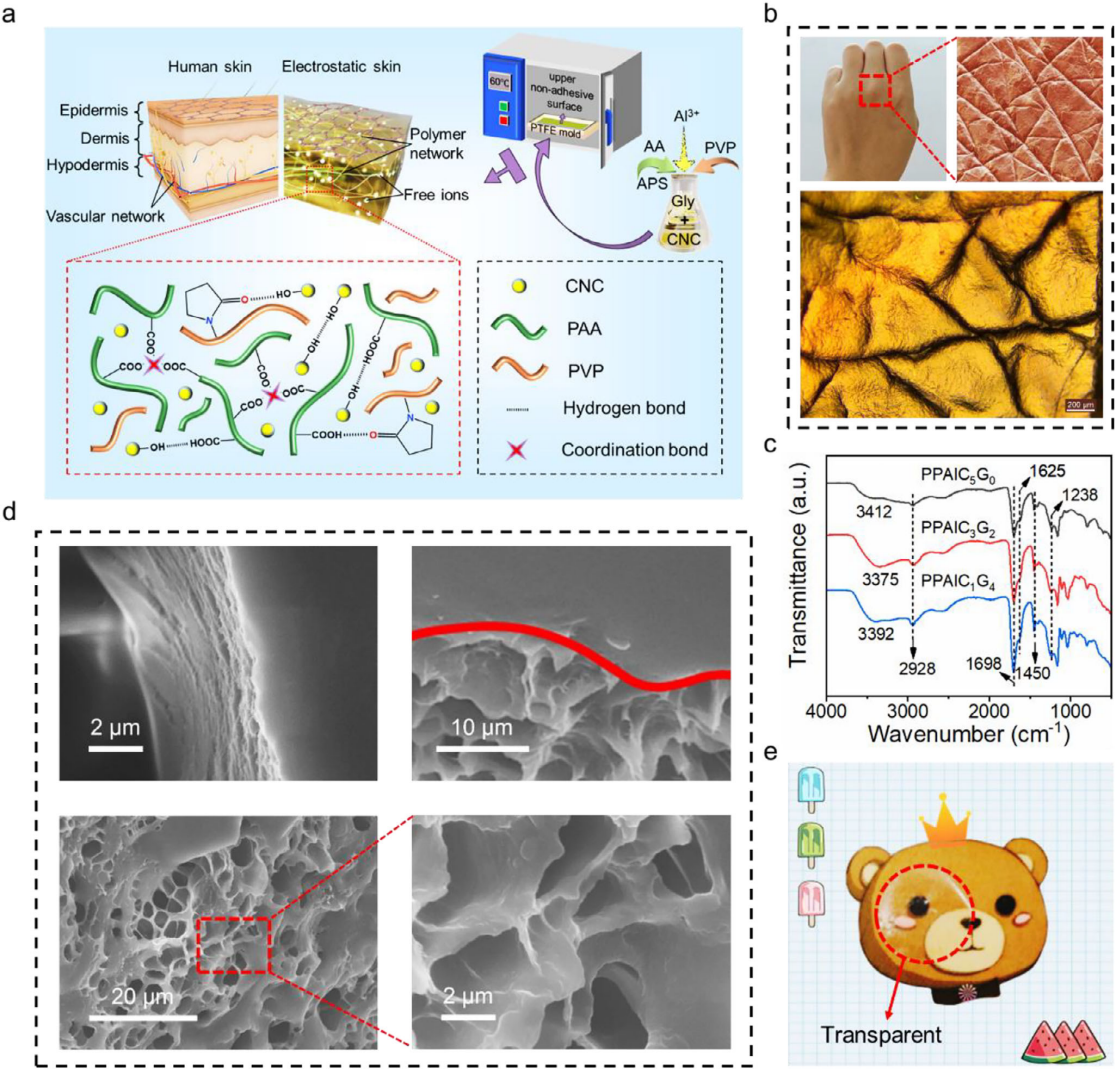
a) Schematic illustration of the design and fabrication of PPAlC_x_G_y_ hydrogel. b) Surface microphotograph of human skin (upper) and PPAlC_x_G_y_ hydrogel (lower). c) FTIR spectra of PPAlC_x_G_y_ hydrogels. d) Overall and amplified cross‐sectional morphologies of PPAlC_x_G_y_ hydrogel. e) Digital photograph of a PPAlC_x_G_y_ hydrogel film demonstrating its optical transparency.

**Figure** **1**a shows the manufacturing process of the PPAlC_x_G_y_ hydrogel via the simple one‐step radical polymerization method. Inspired by the human skin, a high‐performance bionic skin structure was constructed via the interaction among polyacrylic acid (PAA), polyvinylpyrrolidone (PVP), and glycerol (Gly), CNC, and AlCl_3_·6H_2_O. In detail, PAA chains were synthesized by the thermal initiation polymerization of AA monomers, which were entangled through coordination and hydrogen bonding interactions with Al^3+^ and carbonyl groups of PVP, forming a stable 3D network structure. The hydroxyl groups on CNC provide numerous active sites for the formation of hydrogen bonds, including the intramolecular interactions and intermolecular interactions with PAA and PVP, which are able to further enhance the physical and mechanical properties of hydrogels.^[^ ^47^, ^48^ ^]^ In addition, the mechanical strength, flexibility, and transparency of PPAlC_x_G_y_ hydrogel can be readily tuned to satisfy various application demands by modulating the mass ratio of CNC to Gly. For instance, as the ratio of CNC to Gly changes from 5:0 to 1:4, the hydrogel exhibits a gradual transition from white to transparent (Figure S1, Supporting Information). At the same time, the skin‐like texture on the surface of the hydrogel gradually changes from dense to large and loose. The microphotograph in Figure 1b displays that the corrugated surface appears to be highly consistent with the appearance of human skin. In addition, the transparent or white base color of this material also provides convenience for dyeing. As shown in Figure S2 (Supporting Information), the gel can be made into various colors to suit different application scenarios, and the addition of dye does not affect the surface texture, structure, and mechanical properties of the hydrogel material.

The as‐prepared PPAlC_x_G_y_ hydrogels were characterized by FTIR spectra in Figure 1c. All the spectral peaks are almost similar, with differences in intensity that depend on the composition of the components. The broad band ≈3392 cm^−1^ is assigned to the stretching vibration of hydroxyl groups (−OH). The characteristic peak at 1698 cm^−1^ is attributed to the carbonyl absorption band (−C═O) stretching vibration, which reveals the skeleton structure of PAA. The similar results were drawn from the band of 1699 cm^−1^ in Raman spectra (Figure S3, Supporting Information) of PPAlC_x_G_y_ hydrogels. The bands at 1238, 1450, and ≈2928 cm^−1^ are ascribed to C‐N stretching vibrations, C─H bending vibrations, and C‐H stretching vibrations, respectively, which agree with the characteristic absorption peaks of PVP.^[^ ^49^, ^50^ ^]^ These bands were also found in the Raman spectra (1243, 1452, and ≈2928 cm^−1^), which also confirmed the existence of PVP in PPAlC_x_G_y_ hydrogels. In addition, the absence of a vibrational band attributable to the ─C═C─ group at 1612 cm^−1^ indicates that the hydrogel was formed through free‐radical polymerization with no detectable residual monomers.^[^ ^51^ ^]^ Furthermore, the stretching vibration of the ‐OH group exhibits a slight red shift, shifting from 3412 cm^−1^ (PPAlC_5_G_0_) to 3375 cm^−1^ (PPAlC_3_G_2_) and 3392 cm^−1^ (PPAlC_1_G_4_), indicating the presence of hydrogen bonding interactions.^[^ ^52^ ^]^ Moreover, the characteristic peak at 1625 cm^−1^ is assigned to the characteristic asymmetric stretching vibration of ionized carboxyl groups, which indicates the formation of coordination bonds between Al^3+^ and carboxyl groups of PAA.^[^ ^53^ ^]^ Similar results were observed from the band of 1631  cm^−1^ in Raman spectra.

The XRD pattern of the PPAlC_x_G_y_ hydrogels with various proportions of CNC to Gly (CNC:Gly = 5:0, 3:2, 1:4) shows diffraction peaks ≈20.8° and 35.1° (Figure S4, Supporting Information), which are ascribed to the characteristic peak of cellulose.^[^ ^54^ ^]^ The overall broad and weak diffraction peaks indicate that the gels are an amorphous structure, and this low‐crystallinity structure contributes to the enhanced transparency of the material by effectively reducing light scattering at crystalline interfaces.^[^ ^55^ ^]^ Figure 1d displays the cross‐sectional morphology of the PPAlC_x_G_y_ hydrogel, revealing a distinct multilayered architecture characterized by a smooth surface and a heterogeneous interior. A marked structural contrast exists between the surface‐compact thin layer and the interconnected porous network within the bulk phase. Quantitative pore size analysis (Figure S5, Supporting Information) confirms a predominant pore diameter distribution of 1–3 µm, which is consistent with the observed microstructural features. Elemental mapping via energy‐dispersive spectrometry (EDS, Figure S6, Supporting Information) further verifies the homogeneous spatial distribution of C, O, and Al throughout the hydrogel matrix. Notably, the PPAlC_x_G_y_ hydrogels not only exhibit excellent moldability into various complex shapes (Figure S7, Supporting Information) but can also be fabricated into thin, transparent films (Figure 1e). As shown in the digital image, the hydrogel film shows clear visibility, which is attributed to the uniform distribution of polymer chains. The tunable optical transparency is also conducive for applications such as wearable and transparent biomedical devices.

### 2.2 Asymmetric Adhesion Property

**Figure 2:**
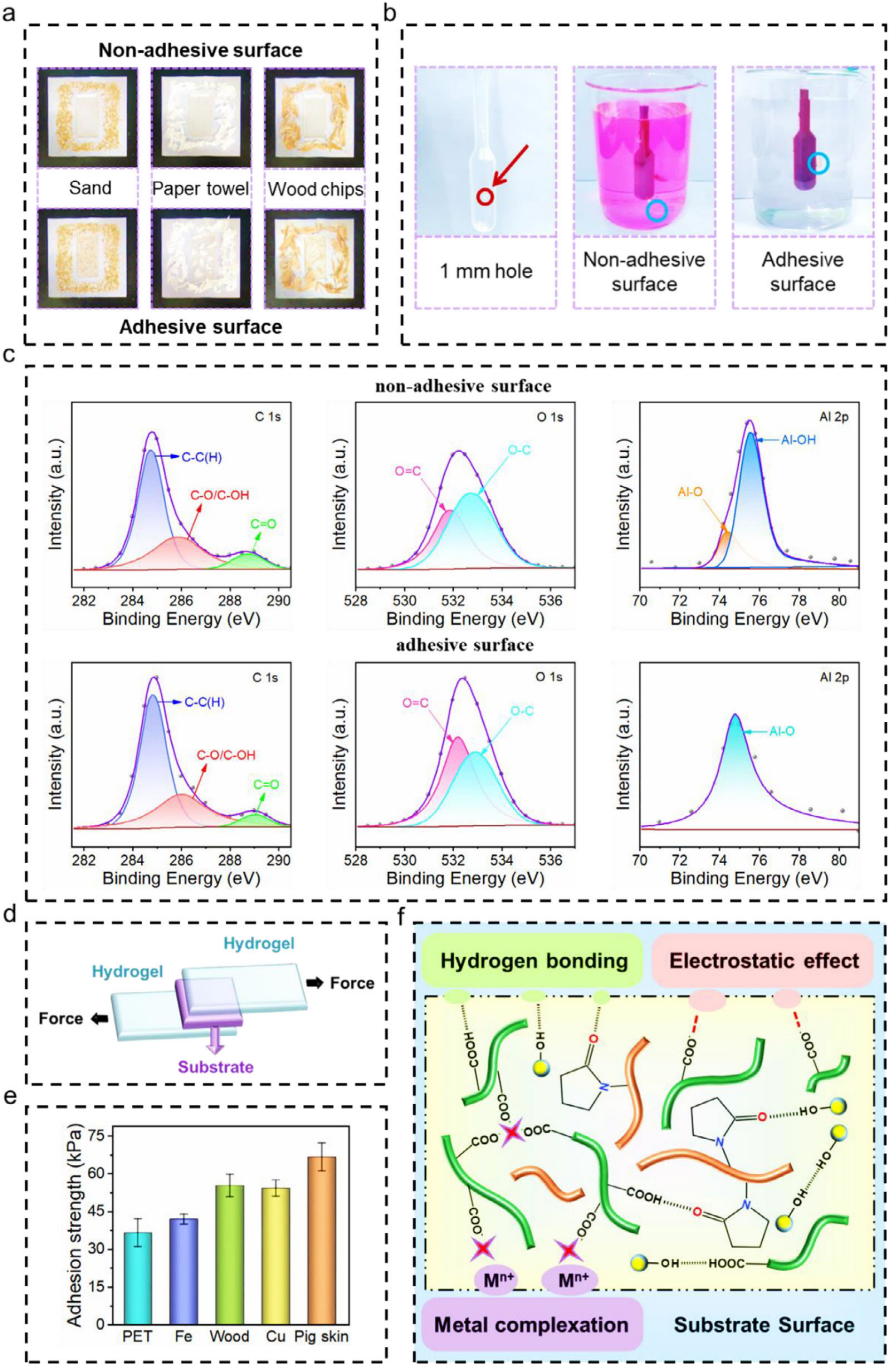
Adhesion properties of PPAlC_x_G_y_ hydrogel. a) Schematic illustration of the adhesion behavior of sand, paper towel, and wood chips on the asymmetric adhesive hydrogel. b) Different repair performance of the asymmetric adhesive PPAlC_x_G_y_ hydrogels for disposable plastic droppers filled with Rhodamine B solution. c) XPS spectra of the adhesive surface and nonadhesive surface of PPAlC_x_G_y_ hydrogel: C 1s and O 1s and Al 2p regions. d) Illustration of adhesion tests. e) Adhesion strength of the PPAlC_x_G_y_ hydrogels on different substrates. f) Schematic diagram of the possible adhesion mechanism between the hydrogel and diverse substrates.

The as‐prepared PPAlC_x_G_y_ hydrogels show a remarkable asymmetric Janus adhesion property. The as‐prepared PPAlC_x_G_y_ hydrogels show a remarkable asymmetric Janus adhesion property. As illustrated in **Figure** **2**a, when various substrates such as paper towels, sand, and wood chips were evenly distributed on both the upper and bottom surfaces of the PPAlC_x_G_y_ hydrogels, strikingly different results were observed after the same intensity of wiping. The upper non‐adhesive surface showed negligible adhesion to all tested materials, whereas the bottom adhesive surface exhibited strong adhesion to the substrates. This distinct contrast in adhesive performance between the two surfaces clearly demonstrates the asymmetric Janus adhesion property of the PPAlC_x_G_y_ hydrogels. In addition, the nonadhesive surface of PPAlC_x_G_y_ hydrogel fails to adhere on the hole of the plastic dropper as illustrated in Figure 2b, leading to the leakage of the Rhodamine B solution. In contrast, the adhesive surface of PPAlC_x_G_y_ hydrogel successfully mended the hole and prevented the Rhodamine B solution from leaking. This indicates that the adhesive surface of the PPAlC_x_G_y_ hydrogel possesses excellent adhesion properties even under water.

The XPS spectra were used to demonstrate the distinction between adhesive surface and non‐adhesive surface and the full spectrum is listed in Figure S8 (Supporting Information). As illustrated in Figure 2c, the C 1s spectra revealed three peaks at ≈284.8, 285.9, and 288.8 eV, corresponding to C─C, C─O/C─OH, and C═O bonds, respectively. The peaks in the O 1s spectra at ≈532.0 and 532.8 eV were attributed to O═C and O─C moieties.^[^ ^56^ ^]^ Compared with the adhesive surface, the signal of C─O/C─OH on the nonadhesive surface exhibits an increased intensity both in the C 1s and O 1s spectra. It is speculated that the surface dehydration induces a condensed surface structure with strengthened hydrogen bonds and coordinate interactions, which enhances the detection concentrations and led to the proportion increase of C─O/C─OH signals. Meanwhile, the Al 2p peak switched from a single peak of adhesive surface to a doublet at 74.3 and 75.5 eV for nonadhesive surface. The 75.5 eV belongs to the coordinate interactions derived from the Al^3+^ and C═O/─OH groups. Whereas the 74.3 Ev, which can be obviously observed on the non‐adhesive surface, is attributed to the oxidation of Al ions (Al‐O).^[^ ^57^ ^]^ It is reasonable as the dense surface layer was dried in the atmosphere, which resulted in the oxidation of Al element and simultaneously contribute to the non‐adhesive property of the upper surface.

The XPS finding is consistent with the FTIR result that a coordination‐sensitive band appears at 1625 cm^−1^, further confirming the formation of a dense surface via the dehydration‐induced crosslinking reorganization of hydrogen bond and Al^3^⁺‐carboxyl coordination occurred on the non‐adhesive surface. The dehydration drives polymer chains to reorganize into a tightly crosslinked network, leading to reduced surface free energy and the burial of adhesive moieties, such as C─O/C─OH and C═O groups (hydroxyl and carboxyl groups). Meanwhile, water loss triggers a transition from a soft viscous state to a stiff brittle state, which leads to the disappearance of adhesion on the upper surface.^[^ ^58^ ^]^ SEM analysis (Figure 1d) also reveals a porous polymeric network inner the hydrogel but a dense and smooth layer on the surface. Combined with the XPS and FTIR, the synergistic analysis of these three characterizations conclusively elucidates the origin of differential adhesion on the upper surface. Furthermore, EDS analysis (Figure S9, Supporting Information) of both the upper non‐adhesive and the bottom adhesive surfaces of the PPAlC_x_G_y_ hydrogels was conducted. The upper textured non‐adhesive surface exhibits a stronger Al signal compared to the flat bottom adhesive surface, which may due to dehydration‐induced surface densification. This observation provides additional evidence for the asymmetric Janus adhesion structure of the PPAlC_x_G_y_ hydrogel.

Moreover, the macroscopic peeling experiments were conducted on human skin to evaluate the adhesive properties of the adhesive surface of PPAlC_x_G_y_ hydrogel. After the rectangular piece of PPAlC_x_G_y_ hydrogel was removed from the skin, no residual adhesion or visible traces were observed on the epidermal surface. Notably, the peeled PPAlC_x_G_y_ hydrogel could repeatedly adhere to skin, and the PPAlC_x_G_y_ hydrogel maintained its structural integrity even after dozens of adhesion and peeling tests (Figure S10, Supporting Information). Furthermore, the PPAlC_x_G_y_ hydrogel adhered easily to gloved fingers and remained firmly attached while bent underwater (Figure S11, Supporting Information). Additionally, the adhesive side of PPAlC_x_G_y_ hydrogels displayed robust adhesion to diverse substrates, such as zinc, aluminum, copper, iron, slide glass, latex glove, fabric, plastic, paper, wood, PET film, and skin tissue in Figure S12 (Supporting Information), indicating the strong adhesion universality of the adhesive side of PPAlC_x_G_y_ hydrogels. A lap‐shear test was conducted on a universal testing machine to further assess the adhesion capability of the adhesive surface of PPAlC_x_G_y_ hydrogels regarding different substrates, as illustrated in Figure 2d. It was noticeable that the highest adhesion strength was obtained on pig skin (66.7 kPa), followed by wood (55.3 kPa), copper (54.3 kPa), iron (42.0 kPa), and PET (36.7 kPa) (Figure 2e). The adhesion ability of PPAlC_x_G_y_ hydrogels on various substrates is attributed to the synergistic cooperation of multiple bonds such as hydrogen bonding, metal complexation, and electrostatic interaction, as shown in Figure 2f.

### 2.3 Mechanical Strength, Self‐Healing Property, and Biocompatibility

**Figure 3:**
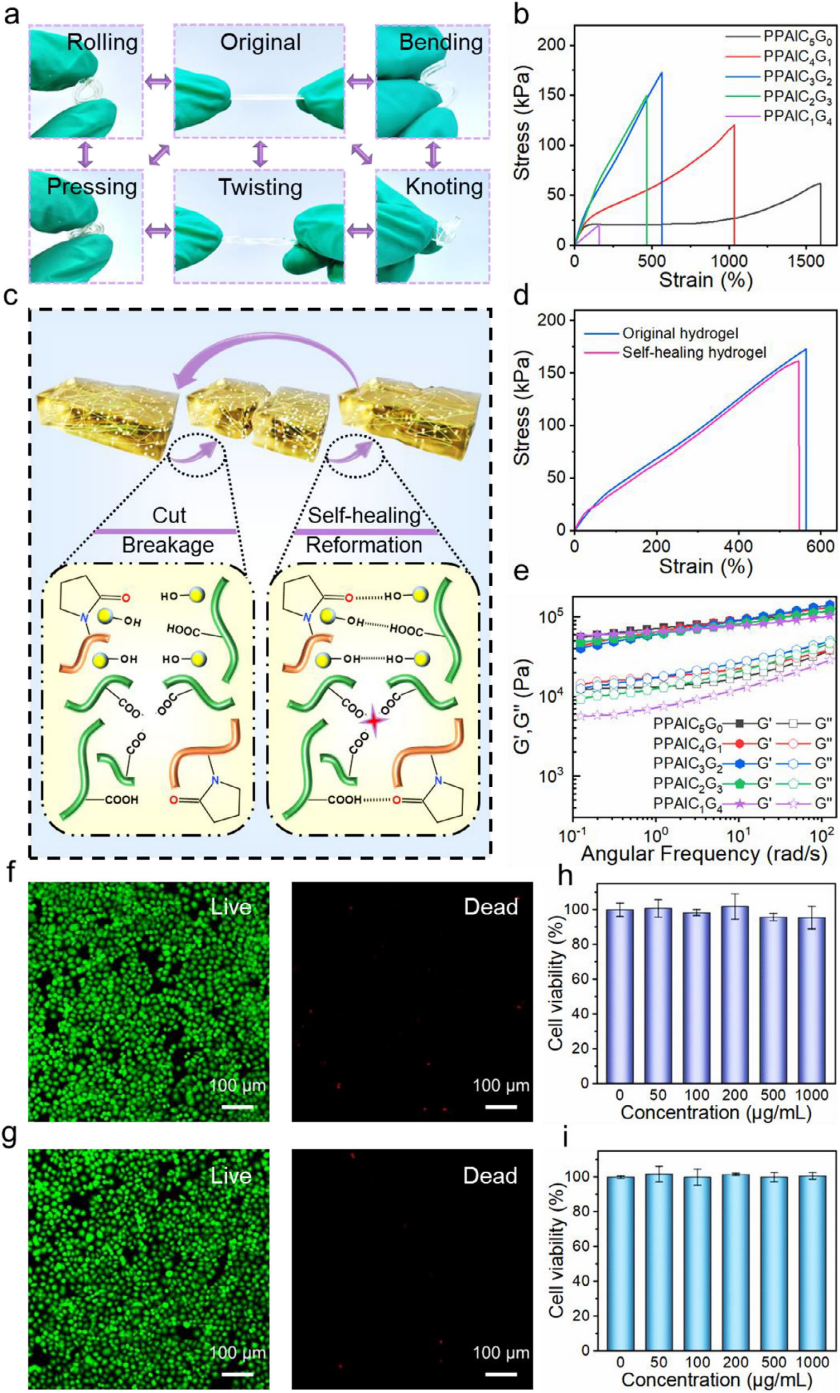
a) Deformation of PPAlC_x_G_y_ hydrogels. b) Tensile stress‐strain curves. c) Schematic diagram of self‐healing mechanism. d) Tensile strain‐stress curves of the original hydrogel and the self‐healed hydrogel. e) Angular frequency dependence of G' and G””. Live/Dead staining morphology of L929 cells for f) PPAlC_3_G_2_ and g) PPAlC_5_G_0_ hydrogels. Cell viability of L929 cells for h) PPAlC_3_G_2_ and i) PPAlC_5_G_0_ hydrogels.

The PPAlC_x_G_y_ hydrogels exhibit remarkable mechanical performance. **Figure** **3**a shows that the PPAlC_x_G_y_ hydrogel is flexible and tough enough to bear various deformations like twisting, bending, knotting, rolling, and pressing, and it can recover to its original shape after the external force is released. Tensile tests were used to further assess the mechanical properties of the PPAlC_x_G_y_ hydrogel via a universal testing machine. As presented in Figure 3b, the maximum tensile strength of 173 kPa was obtained on PPAlC_3_G_2_ hydrogel (CNC:Gly = 3:2), which is 10.1 times higher than that of the PPAlC_1_G_4_ hydrogel. Meanwhile, the highest tensile strain of 1593% is ascribed to PPAlC_5_G_0_ hydrogel, which is 8.7 times higher than that of the PPAlC_1_G_4_ hydrogel. However, the incorporation of excess soft component Gly leads to a rapid fracture during the stretching of PPAlC_1_G_4_ hydrogels. The enhancement of mechanical properties was dominantly ascribed to strong hydrogen bonding and electrostatic interactions between carboxyl and hydroxyl groups on PAA, PVP, and CNC. Moreover, the carboxyl groups on PAA coordinate with Al^3+^ in the matrix, which also effectively improves the physical strength of the PPAlC_x_G_y_ hydrogel. Besides, the evenly distributed CNCs reinforce PPAlC_x_G_y_ hydrogels by forming physically cross‐linked networks that restrict polymer chain mobility and amplify crosslinking density, while their rigid structure redistributes stress and dissipates energy through interfacial slippage and crack deflection, synergistically enhancing both stiffness and toughness of the polymer matrix.^[^ ^59^, ^60^ ^]^

In addition to superior mechanical performance, the self‐healing ability is important to enhance the reliability and extend the service life of hydrogel devices. As shown in Figure 3c, when PPAlC_x_G_y_ hydrogel was cut into two pieces, the hydrogen bonds and coordination bonds underwent breakage. While the two pieces of hydrogels were kept in contact with each other at room temperature, they could be recombined as an integral hydrogel through dynamic cross‐linking and the reformation of abundant hydrogen bonds, which proved that the hydrogel has self‐healing properties. The reversible coordination bonds between carboxyl groups of PAA and Al^3+^ in the system also contribute to the self‐healing property of the hydrogel. The tensile stress‐strain curves of PPAlC_x_G_y_ hydrogels are measured to further investigate the self‐healing ability. It is noteworthy that the healing efficiency of PPAlC_3_G_2_ hydrogel remarkably reached 93.5% after 6 h as shown in Figure 3d, proving an outstanding self‐healing property.

Dynamic oscillatory rheology tests were carried out to gain insight into the viscoelasticity of PPAlC_x_G_y_ hydrogels. For strain sweep in Figure S13a (Supporting Information), G″ of PPAlC_x_G_y_ hydrogels was lower than G′ at very low strain amplitudes, which was consistent with the existence of a network structure.^[^ ^61^ ^]^ As shown in Figure S13b (Supporting Information), the gelatinization threshold parameter (tan δ) is consistently below 1 over the entire frequency range, which indicates that a solid hydrogel has formed.^[^ ^62^ ^]^ Moreover, Figure 3e shows the rheological frequency scanning of the PPAlC_x_G_y_ hydrogels. Notably, the PPAlC_x_G_y_ hydrogels with different proportions of CNC to Gly exhibit higher rheological G′ than G″ over the whole angular frequency (ω) range, indicating the elastic network character inner the hydrogels.^[^ ^63^ ^]^

Biocompatibility is essential for hydrogels used as wearable sensors, as they prevent adverse reactions and maintain the comfort and safety for users over a prolonged service life. The biocompatibility of PPAlC_x_G_y_ hydrogels was evaluated by 3‐(4,5‐dimethylthiazol‐2‐yl)‐2,5‐diphenyltetrazolium bromide (MTT)igu assay. L929 cells were cultivated on the PPAlC_3_G_2_ and PPAlC_5_G_0_ hydrogels, and the live/dead staining morphology of L929 cells was investigated under a laser confocal microscopy in Figure 3f,g, respectively. After 24 h of incubation, a lot of green fibroblasts were observed and the L929 cells were distributed evenly in the entire area for both PPAlC_3_G_2_ and PPAlC_5_G_0_. Compared with the 100% cell viability of control group, the L929 cell viability with a hydrogel concentration of 1000 µg mL^−1^ still maintains up to 100.7% (Figure 3h) and 99.5% (Figure 3i) for PPAlC_3_G_2_ and PPAlC_5_G_0_, respectively, which indicates that the prepared hydrogels conduce to the growth and proliferation of L929 cells and have excellent biocompatibility.^[^ ^64^ ^]^ These results suggested that the PPAlC_3_G_2_, which possesses superior mechanical properties as illustrated in Figure 3b, demonstrates enhanced biocompatibility simultaneously compared with PPAlC_5_G_0_.

### 2.4 Electromechanical Properties and Sensing Applications

**Figure 4:**
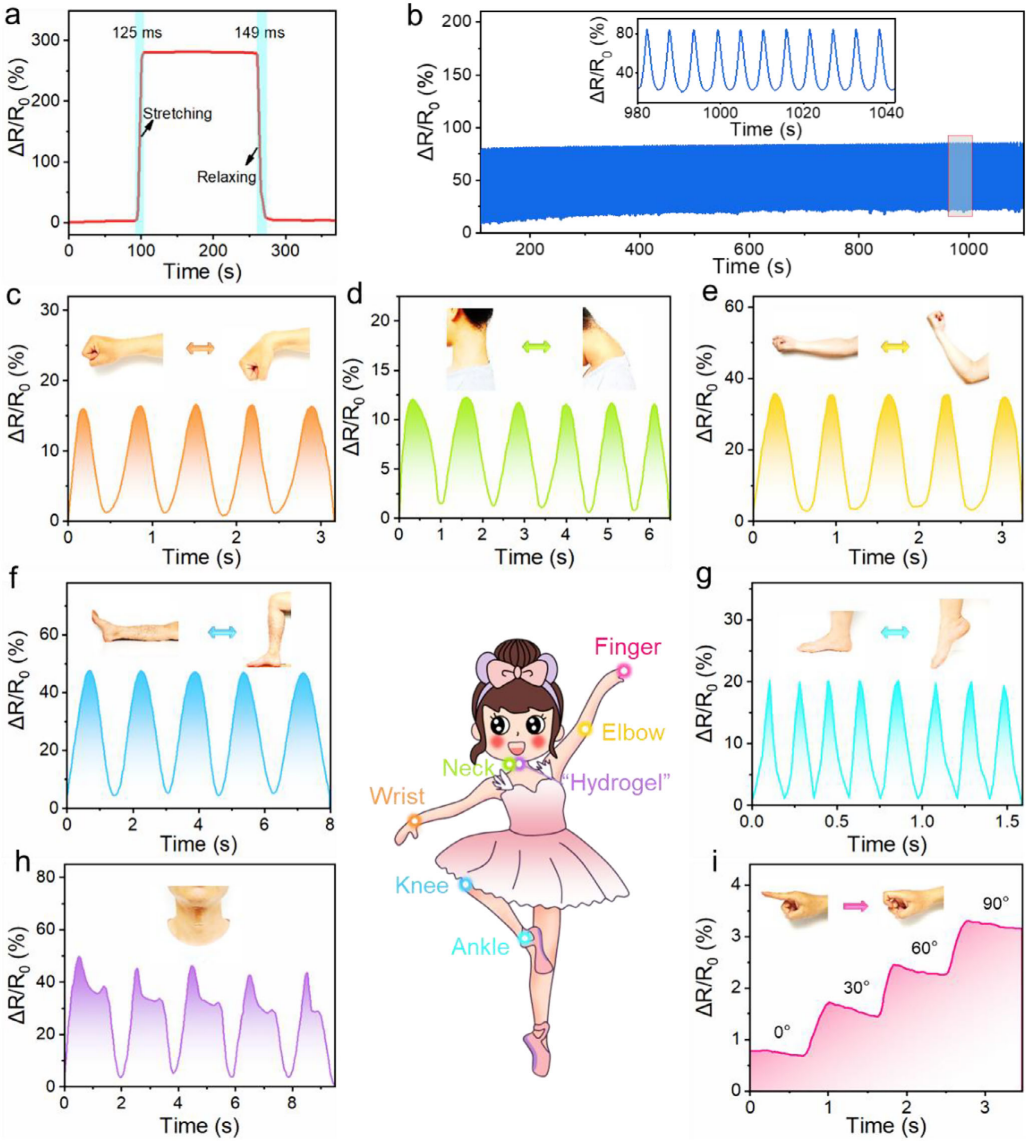
Electromechanical properties of PPAlC_x_G_y_ hydrogels. a) Response and recovery times. b) Cyclic stability test over 2000 loading and unloading cycles. Real‐time motion monitoring. c) Wrist bending, d) neck bending, e) elbow bending, f) knee bending g) ankle bending, h) speaking and i) finger bending with certain angles.

The PPAlC_x_G_y_ hydrogel, featuring exceptional properties such as high strength, stretchability, self‐healing ability, excellent tensile and bending toughness, along with appropriate ion concentration, shows great promise for applications in wearable devices, particularly as a soft sensor. Its interconnected micron‐scale porous structure facilitates ion transport within the multi‐layered network, enabling the PPAlC_x_G_y_ hydrogel to function as an ionic gel sensor with excellent strain sensitivity. As shown in Figure S14 (Supporting Information), the conductivity of the PPAlC_x_G_y_ hydrogels were found to increase from 0.04 to 0.38 mS cm^−1^ when the ratio of CNC to Gly was adjusted from 5:0 to 1:4. An effective body monitor sensor for wearability should possess fast response, real‐time data collection, and stable characteristics to ensure reliable performance.^[^ ^65^, ^66^ ^]^ As presented in **Figure** **4**a, the PPAlC_x_G_y_ hydrogel sensor offered rapid loading response (0.13 s) and unloading recovery abilities (0.15 s), which showed an excellent response capability as a bionic skin. In addition, Figure 4b and the enlarged inset reveal that the stable signal output can be sustained after multiple cycles, which is beneficial for the stability and reusability of the PPAlC_x_G_y_ hydrogel sensor during its service life.

In addition, the sensitivity of the hydrogel strain sensor is an important indicator. As shown in Figure S15 (Supporting Information), the sensitivity curve calculated according to Equation (S3, Supporting Information) was divided into three regions with different slopes, corresponding to three different gauge factor (GF) values. The GF changed from 1.09 to 1.93 and 3.2 when the hydrogel strain ranges increased from 0% to 100%, from 100% to 200% and from 200% to 500%, respectively, enabling precise detection across physiological and mechanical ranges. This behavior suggests that the sensor is capable of accurately detecting both subtle and large‐scale deformations. Notably, these findings surpass both the sensitivity and detection range reported in some of the literature studies (Figure S16 and Table S2, Supporting Information).^[^ ^67^, ^68^, ^69^, ^70^, ^71^, ^72^, ^73^, ^74^, ^75^, ^76^ ^]^ As shown in Table S2 (Supporting Information) and the radar chart in Figure S16 (Supporting Information), the PPAlC_x_G_y_ hydrogel‐based sensor exhibits competitive performance when compared with the state‐of‐the‐art hydrogel sensors. In particular, the combination of high sensitivity (GF reaches up to 3.20), wide strain detection range (0–500% strain), fast response (0.13 s), and self‐adhesion without the need for external fixation offers outstanding advantages for practical wearable applications. Additionally, the one‐step molding process for creating an asymmetric Janus structure with textures further enhances its performance and ease of use.

In order to assess their sensing ability in practical applications, the PPAlC_x_G_y_ hydrogels were applied to multiple regions on the human body to monitor motion signals. The detailed configuration of the sensing test setup is illustrated in Figure S17 (Supporting Information), including conformal adhesion of the hydrogel sensor to human joints, its connection to the signal acquisition module, and real‐time data recording workflow during motion monitoring. Figure 4c illustrates the relative resistance change curves of the wrist area during movement. Notably, when the human wrist was held at a certain bending angle, the resulting electrical signals remained clear and stable, indicating the exceptional stability of the PPAlC_x_G_y_ hydrogel as a sensor. Moreover, the detection of neck movement was also demonstrated in Figure 4d. Specifically, a significant signal rise was observed when the neck bends, and it recovered to the original level when the neck joint was totally straightened, which reveals that the PPAlC_x_G_y_ hydrogel sensor possesses stable sensing, fast response, and excellent self‐recovery performance. Besides, Figure 4e–g presents the real‐time resulting curves of the relative resistance changes of the PPAlC_x_G_y_ hydrogel sensor attached to the elbow, knee, and ankle joints, respectively. Since the gel sensors have different elongation rates in the bending direction at various parts of the body, the amplitude of the generated signals varies significantly. Notably, the PPAlC_x_G_y_ hydrogel sensor still maintains excellent sensing accuracy and stability at different body parts.

In addition to detecting large‐scale movements on various parts of the body, the PPAlC_x_G_y_ hydrogel sensor is also capable of sensing subtle motion signals, such as speaking and finger bending. As shown in Figure 4h, the relative resistance change curves exhibited stable and regular trends during the periodic saying “Hydrogel,” showing great promise of the PPAlC_x_G_y_ hydrogel for phonation rehabilitation. Significantly, the finger bending motion with different bending angles could also be detected. As the bending angle gradually increased from 0° to 90° in Figure 4i, the relative resistance of the PPAlC_x_G_y_ hydrogels was regularly increased, demonstrating the accurate and transient sensing of finger movements. The consistent signal periodicity/amplitude during cyclic stability test and human motion monitoring proves the durability of PPAlC_x_G_y_ hydrogels. All of the above results demonstrate that the PPAlC_x_G_y_ hydrogels sensor possesses rapid response and prominent sensing ability, which makes it an ideal candidate for human activity and health monitoring. In addition, electromechanical measurements were carried out at 40%, 60%, and 80% relative humidity (RH) under room temperature to evaluate the influence of environmental humidity on sensor performance. As shown in Figure S18 (Supporting Information), the resulting resistance change profiles are nearly overlapping across all tested humidity levels, indicating little impact of humidity on output characteristics. This consistency under varying humidity conditions highlights the PPAlC_x_G_y_ hydrogel's suitability for reliable operation in wearable applications. Notably, Figure S19 (Supporting Information) shows that an electronic clock can display time when powered by the sensor device, demonstrating the potential of the hydrogel as a self‐powered sensing platform based on the primary battery principle.

### 2.5 Flexible Sensors Applications for Human–Machine Interaction

Owing to its unique combination of Janus adhesion, high conductivity, and a surface that emulates the texture of human skin, the PPAlC_x_G_y_ hydrogel shows great potential as a skin electrode for electrophysiological signal detection. This material can be efficiently fabricated into sensors tailored to detect crucial biological signals, including electroencephalogram (EEG) and electromyography (EMG) signals. The excellent adhesion capability enables the formation of stable interfacial contact with skin tissue, thereby effectively mitigating motion artifacts during dynamic measurements. Furthermore, its optimized electrical conductivity ensures reliable signal acquisition with high fidelity, meeting the requirements for precise bio‐signal monitoring.^[^ ^77^, ^78^ ^]^ Notably, the biomimetic skin‐textured surface morphology of hydrogel not only achieves realistic cutaneous appearance but also minimizes visual mismatch with natural epidermis, thereby improving the aesthetic integration with human skin.

**Figure 5:**
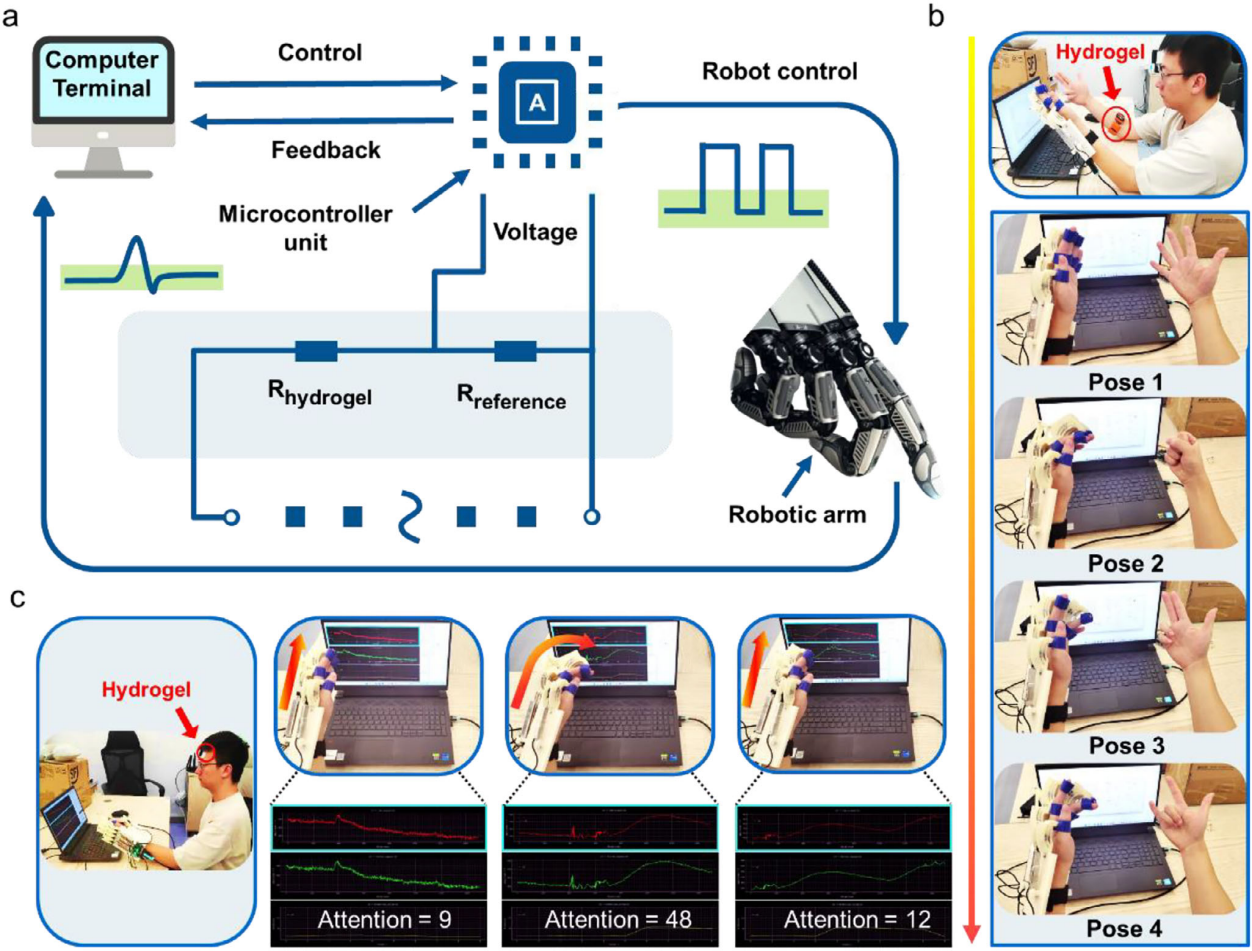
a) Control diagram of the assistive robotic arm. b) EMG signals recognition from different hand gestures. c) A feedback control system based on EEG signals to control the assistive robotic arm.

To ensure optimal performance and user interaction, an integrated closed‐loop control system was established for the assistive robotic arm (**Figure** **5**a), with a specific focus on force‐feedback mechanisms that can monitor and respond to force inputs in real‐time. The proposed system employs the right hand as the intact limb to perform gestural motions while maintaining the left hand stationary to simulate a motor‐impaired condition. During gesture execution, EMG signals captured by the electronic skin are transmitted to the STM32 microcontroller. The acquired signals undergo analysis and noise reduction before being converted into output commands that drive a robotic arm to replicate corresponding gestures. Through this mechanism, the motor‐impaired limb achieves desired motions via robotic assistance, thereby facilitating movement restoration for individuals with motor disabilities. As shown in Figure 5b and further visualized in Movie S1 (Supporting Information), when the gesture altered from pose 1 to pose 4, the assistive robotic arm correspondingly executes an identical transformation, showcasing the ability to synchronize its movements with the given gesture changes.

In addition, the EEG signals can be collected and conducted by the e‐skin to motivate assistive robotic arm. Figure 5c and Movie S2 (Supporting Information) provide a detailed depiction of the activation sequence for the assistive robotic arm, including an integrated feedback control system. Specifically, these collected signals undergo sequential processing and are ultimately converted into output commands, where fluctuations in attention scores serve as the key feedback mechanism to drive real‐time operation of the assistive robotic arm. The attention score is correlated with the degree of the brain's craving. The increased desire to control the robotic arm causes increased EEG intensity and attention score. In order to reduce the impact of signal noise, the attention score was set to 20. When the attention score is below the pre‐defined value of 20, the assistive robotic arm remains in a stationary state. The assistive robotic arm starts its operation when the attention score exceeds 20, and it returns to the original state while the brain stops the mind of “motivate the arm.” The textured Janus hydrogel serves as an e‐skin well facilitate the human–machine interfacing combined with an outstanding aesthetic compatibility, which presents a transformative potential for hydrogel‐based bio‐interfaces in continuously expanding human–machine symbiosis.

## 3 Conclusion 

In summary, inspired by the natural Janus adhesion and texture‐forming properties of Chinese Nian Gao, this study successfully developed a bio‐hydrogel‐based electronic skin with asymmetric adhesion and biomimetic surface textures through a facile one‐pot fabrication strategy. The e‐skin, constructed via hydrogen bonding and metal‐ligand coordination, exhibits favorable mechanical properties (tensile strength of 173 kPa, strain of 1593%) and asymmetric adhesion property. The e‐skin achieved strong interfacial bonding (66.7 kPa) on the bottom surface for stable signal acquisition, meanwhile, maintained a nonadhesive upper surface for preventing external interference. The differential adhesion of the hydrogel ensures its reliable performance in dynamic utilizations and omits the vital steps, such as encapsulation and postprocessing in the preparation of common wearable electronics. Additionally, the skin‐like surface textures enhance visual authenticity for applications such as skin coverage and humanoid robot encapsulation. The hydrogel also demonstrates outstanding biocompatibility and a high self‐healing efficiency (93.5%), which further supports its suitability for long‐term wearable use. As a flexible sensor, the hydrogel was able to monitor body movements, and a real‐time assistive robotic arm can be well activated via EMG and EEG signals. This work bridges biomimetic design with advanced material engineering, offering a scalable and versatile platform for next‐generation wearable electronics. The integration of aesthetic appeal, mechanical robustness, and sensing functionality positions this Janus hydrogel as a promising candidate for future applications in personalized healthcare, adaptive human–machine interfaces, and soft robotics.

## Conflict of Interest

The authors declare no conflict of interest.

## Ethics Approval Statement

The human research in this study was prospectively reviewed and approved by the ethics committee (Ethics Approval 2022‐(ER)‐047) of Haikou People's Hospital, China. The human research was performed on subjects who provided informed consent before participating. We certify that the study was performed in accordance with the 1964 Declaration of HELSINKI and later amendments.

## Supporting information



Supporting Information

Supplemental Movie 1

Supplemental Movie 2

## Data Availability

The data that support the findings of this study are available from the corresponding author upon reasonable request.

## References

[1] Y. Pei , J. An , K. Wang , Z. Hui , X. Zhang , H. Pan , J. Zhou , G. Sun , Small 2023, 19, 2301884. 10.1002/smll.202301884 37162447

[2] Y. Wang , M. Tebyetekerwa , Y. Liu , M. Wang , J. Zhu , J. Xu , C. Zhang , T. Liu , Chem. Eng. J. 2021, 420, 127637.

[3] L. Wu , Y. Kang , X. Shi , B. Yuezhen , M. Qu , J. Li , Z.‐S. Wu , ACS Nano 2023, 17, 13522. 37439503 10.1021/acsnano.3c01976

[4] Z. Dai , K. Feng , M. Wang , M. Lei , S. Ding , J. Luo , Q. Xu , B. Zhou , Nano Energy 2022, 97, 107173.

[5] X. Dai , Y. Wu , Q. Liang , J. Yang , L.‐B. Huang , J. Kong , J. Hao , Adv. Funct. Mater. 2023, 33, 2304415.

[6] R. Jiang , J. Pu , Y. Wang , J. Chen , G. Fu , X. Chen , J. Yang , J. Shen , X. Sun , J. Ding , X. Xu , Interdiscip. Mater. 2024, 3, 414.

[7] H. Zhao , S. Hao , Q. Fu , X. Zhang , L. Meng , F. Xu , J. Yang , Chem. Mater. 2022, 34, 5258.

[8] P. Manchi , M. V. Paranjape , A. Kurakula , V. S. Kavarthapu , C.‐W. Kim , J. S. Yu , Nano Energy 2025, 142, 111184.

[9] H. Patnam , S. A. Graham , P. Manchi , M. V. Paranjape , J. S. Yu , ACS Appl. Mater. Interfaces 2023, 15, 16768. 36973637 10.1021/acsami.3c00386

[10] H. Patnam , S. A. Graham , P. Manchi , M. V. Paranjape , Y. S. Huh , J. S. Yu , Adv. Compos. Hybrid Mater. 2024, 7, 56.

[11] F. Wang , Y. Xue , X. Chen , P. Zhang , L. Shan , Q. Duan , J. Xing , Y. Lan , B. Lu , J. Liu , Adv. Funct. Mater. 2024, 34, 2314471.

[12] J. Liao , Z. Ma , S. Liu , W. Li , X. Yang , M. E. Hilal , X. Zhou , Z. Yang , B. L. Khoo , Adv. Funct. Mater. 2024, 34, 2401930.

[13] X. Zhang , Y. Su , J. Xu , Y. Jin , H. Zhang , G. Ma , J. Xu , M. Zhou , X. Zhou , F. Cao , Y. Chang , Y. Wang , B. Zhao , S. Yi , J. Chen , D. Fang , X. Lv , L. Liu , Nano Energy 2025, 133, 110484.

[14] C. Tang , K. Zhang , S. Yu , H. Guan , M. Cao , K. Zhang , Y. Pan , S. Zhang , X. Sun , H. Peng , Small 2024, 20, 2405000. 10.1002/smll.202405000 39152934

[15] Y. Han , Y. Liu , Y. Liu , D. Jiang , Z. Wu , B. Jiang , H. Yan , Z. Toktarbay , Adv. Compos. Hybrid Mater. 2025, 8, 154.

[16] S. Liu , W. Qing , J. Zhang , S. Liao , Q. Wang , K. Wei , W. Yan , L. Zhao , H. Zou , Adv. Funct. Mater. 2025, 35, 2419459.

[17] L. Zhu , P. Xu , B. Chang , J. Ning , T. Yan , Z. Yang , H. Lu , Adv. Funct. Mater. 2024, 34, 2400363.

[18] K. Long , Y. Luo , C. Hu , B. Xu , X. Gu , Z. Ding , S. Guo , Chem. Eng. J. 2025, 513, 162828.

[19] Y. Long , B. Jiang , T. Huang , Y. Liu , J. Niu , Z. L. Wang , W. Hu , Adv. Funct. Mater. 2023, 33, 2304625.

[20] R. Zhao , X. Yan , H. Lin , Z. Zhao , S. Song , Chem. Eng. J. 2025, 510, 161645.

[21] M. S. Rahman , A. Shon , R. Joseph , A. Pavlov , A. Stefanov , M. Namkoong , H. Guo , D. Bui , R. Master , A. Sharma , J. Lee , M. Rivas , A. Elati , Y. Jones‐Hall , F. Zhao , H. Park , M. A. Hook , L. Tian , Sci. Adv. 2025, 11. 10.1126/sciadv.ads4415 PMC11927610 40117365

[22] W. Zhang , S. Liu , L. Wang , B. Li , M. Xie , Y. Deng , J. Zhang , H. Zeng , L. Qiu , L. Huang , T. Gou , X. Cen , J. Tang , J. Wang , Carbohydr. Polym. 2024, 344, 122538. 39218556 10.1016/j.carbpol.2024.122538

[23] D. Wang , J. Zhang , C. Fan , J. Xing , A. Wei , W. Xu , Q. Feng , Q. Wei , Compos. Part B Eng. 2022, 243, 110116.

[24] Y. Cui , J. Hu , Z. Dong , B. Li , C. Chang , Nat. Commun. 2025, 16, 3384. 40204743 10.1038/s41467-025-58731-4 PMC11982279

[25] W. Wei , W. Liu , H. Kang , X. Zhang , R. Yu , J. Liu , K. Huang , Y. Zhang , M. Xie , Y. Hu , H. Dai , Adv. Healthcare Mater. 2023, 12, 2300108. 10.1002/adhm.202300108 36763493

[26] B. Dúzs , O. Skarsetz , G. Fusi , C. Lupfer , A. Walther , Nat. Commun. 2024, 15, 8957. 39419997 10.1038/s41467-024-53368-1 PMC11487081

[27] C. Rinoldi , Y. Ziai , S. S. Zargarian , P. Nakielski , K. Zembrzycki , M. A. Haghighat Bayan , A. B. Zakrzewska , R. Fiorelli , M. Lanzi , A. Kostrzewska‐Księżyk , R. Czajkowski , E. Kublik , L. Kaczmarek , F. Pierini , ACS Appl. Mater. Interfaces 2023, 15, 6283. 36576451 10.1021/acsami.2c17025

[28] S. S. Zargarian , C. Rinoldi , Y. Ziai , A. Zakrzewska , R. Fiorelli , M. Gazińska , M. Marinelli , M. Majkowska , P. Hottowy , B. Mindur , R. Czajkowski , E. Kublik , P. Nakielski , M. Lanzi , L. Kaczmarek , F. Pierini , Small Sci. 2025, 5, 2400463. 40395354 10.1002/smsc.202400463 PMC12087770

[29] L. Wang , J. Wei , M. You , Y. Jin , D. Li , Z. Xu , A. Yu , J. Li , C. Chen , Carbohydr. Polym. 2025, 354, 123345. 39978888 10.1016/j.carbpol.2025.123345

[30] Y. Liang , D. Zou , Y. Zhang , Z. Zhong , Chem. Eng. J. 2023, 475, 145928.

[31] X. Liu , X. Ji , R. Zhu , J. Gu , J. Liang , Adv. Mater. 2024, 36, 2309508. 10.1002/adma.202309508 38190548

[32] J. Liu , W. Zhao , Z. Ma , H. Zhao , L. Ren , Mater. Today 2024, 81, 84.

[33] L. Xie , H. Lei , Y. Liu , B. Lu , X. Qin , C. Zhu , H. Ji , Z. Gao , Y. Wang , Y. Lv , C. Zhao , I. Z. Mitrovic , X. Sun , Z. Wen , Adv. Mater. 2024, 36, 2406235. 10.1002/adma.202406235 39007254

[34] J. Li , Q. Chen , S. Li , X. Zeng , J. Qin , X. Li , Z. Chen , W. Zheng , Y. Zhao , Z. Huang , X. Yang , L. Gan , Chem. Eng. J. 2023, 473, 145212.

[35] Y. Xu , Y. Han , Y. Li , J. Li , J. Li , Q. Gao , Chem. Eng. J. 2022, 437, 135437.

[36] C.‐Y. Zou , X.‐X. Lei , J.‐J. Hu , Y.‐L. Jiang , Q.‐J. Li , Y.‐T. Song , Q.‐Y. Zhang , J. Li‐Ling , H.‐Q. Xie , Bioact. Mater. 2022, 16, 388. 35415284 10.1016/j.bioactmat.2022.02.034 PMC8965776

[37] N. Wen , S. Li , H. Jiang , J. Yang , W. Yang , Y. Song , J. Long , J. Zhao , Z. Lin , X. Yu , Y. Wei , S. Lu , X. Huang , T. Zhou , Adv. Funct. Mater. 2024, 35, 2411959.

[38] S. Kondaveeti , G. Choi , S. C. Veerla , S. Kim , J. Kim , H. J. Lee , U. Kuzhiumparambil , P. J. Ralph , J. Yeo , H. E. Jeong , Nano Converg 2024, 11, 12. 38512587 10.1186/s40580-024-00419-4 PMC10957857

[39] S. Li , W. Ahmed , S. Wang , X. Hu , B. Wang , Z. Wang , L. Shen , Y. Zhu , C. Gao , Compos. Part B Eng. 2024, 283, 111668.

[40] M. Zhang , H. An , Z. Gu , Z. Huang , F. Zhang , B.‐G. Jiang , Y. Wen , P. Zhang , Adv. Mater. 2023, 35, 2212015. 10.1002/adma.202212015 37205796

[41] M. Brebu , D. Pamfil , I. Stoica , M. Aflori , G. Voicu , E. Stoleru , Carbohydr. Polym. 2024, 339, 122288. 38823936 10.1016/j.carbpol.2024.122288

[42] X. Gong , C. Fu , N. Alam , Y. Ni , L. Chen , L. Huang , H. Hu , Biomacromolecules 2022, 23, 2272. 35486379 10.1021/acs.biomac.1c01640

[43] I. Kellersztein , D. Tish , J. Pederson , M. Bechthold , C. Daraio , Adv. Mater. 2025, 37, 2413618. 10.1002/adma.202413618 39558799

[44] H. Qiao , S. Sun , P. Wu , Adv. Mater. 2023, 35, 2300593. 10.1002/adma.202300593 36861380

[45] X. Zhao , Z. Zhang , L. Xu , F. Gao , B. Zhao , T. Ouyang , Z. Kang , Q. Liao , Y. Zhang , Nano Energy 2021, 85, 106001.

[46] S. Hou , Q. Huang , H. Zhang , Q. Chen , C. Wu , M. Wu , C. Meng , K. Yao , X. Yu , V. A. L. Roy , W. Daoud , J. Wang , W. J. Li , Adv. Sci. 2024, 11, 2400234. 10.1002/advs.202400234 PMC11425864 38988056

[47] B. Li , Y. Chen , W. Wu , X. Cao , Z. Luo , Carbohydr. Polym. 2023, 317, 121092. 37364960 10.1016/j.carbpol.2023.121092

[48] Y. Li , M. Zhu , R. Yin , G. Li , Y. Fu , M. Qin , Z. Yuan , Carbohydr. Polym. 2025, 361, 123628. 40368555 10.1016/j.carbpol.2025.123628

[49] Y. Cui , Z. Huang , L. Lei , Q. Li , J. Jiang , Q. Zeng , A. Tang , H. Yang , Y. Zhang , Nat. Commun. 2021, 12, 5922. 34635666 10.1038/s41467-021-26237-4 PMC8505635

[50] S. Nayak , S. R. Prasad , D. Mandal , P. Das , J. Hazard. Mater. 2020, 392, 122287. 32066019 10.1016/j.jhazmat.2020.122287

[51] V. M. Möhring , K. Seevogel , G. Fink , Fresenius Z. Für Anal. Chem. 1986, 323, 330.

[52] D. Li , N. Liu , X. Yao , Q. Gou , J. Yue , D. Yang , X. Chen , M. Xiao , Food Hydrocoll. 2023, 139, 108596.

[53] J. Pan , Y. Jin , S. Lai , L. Shi , W. Fan , Y. Shen , Chem. Eng. J. 2019, 370, 1228.

[54] H. Kargarzadeh , I. Ahmad , I. Abdullah , A. Dufresne , S. Y. Zainudin , R. M. Sheltami , Cellulose 2012, 19, 855.

[55] C. He , J. Qi , J. Liao , Y. Song , C. Wu , Food Hydrocoll 2023, 144, 108988.

[56] D. B. Salem , K. Yahiaoui , M. Bernardo , J. M. Gatica , A. Ouakouak , F. Touahra , A. Saad Eltaweil , H. N. Tran , J. Environ. Manage. 2025, 383, 125407. 40273783 10.1016/j.jenvman.2025.125407

[57] W. Xiao , Y. Shao , J. Yu , B. Zhang , H. Shu , Y. Zhang , Sep. Purif. Technol. 2022, 299, 121770.

[58] X. Guo , Y. Gao , J. Yu , S. Qiu , X. Wang , S. Wang , C. Zhang , B. Yi , Y. Gao , Adv. Healthcare Mater. 2025, 2500277, 10.1002/adhm.202500277. 40357898

[59] P. Cui , J. Chen , K. Fu , J. Deng , T. Sun , K. Chen , P. Yin , ACS Appl. Mater. Interfaces 2024, 16, 53022. 39306751 10.1021/acsami.4c13264

[60] C. Duan , B. Wang , J. Li , J. Zeng , D. Cao , J. Xu , W. Gao , K. Chen , Adv. Funct. Mater. 2024, 34, 2315865.

[61] Y. Meng , L. Ye , J. Sci. Food Agric. 2017, 97, 3831. 28150437 10.1002/jsfa.8247

[62] H. Hosseini , A. Zirakjou , V. Goodarzi , S. M. Mousavi , H. A. Khonakdar , S. Zamanlui , Int. J. Biol. Macromol. 2020, 152, 57. 32057868 10.1016/j.ijbiomac.2020.02.095

[63] F. Mazahir , V. Rahi , R. K. Kaundal , M. I. Alam , A. K. Yadav , Chem. Eng. J. 2025, 503, 158197.

[64] N. Zhao , W. Yuan , Compos. Part B Eng. 2022, 242, 110095.

[65] J. W. Nam , C.‐H. Moon , D.‐H. Kim , M. H. Kim , W. H. Park , Chem. Eng. J. 2024, 498, 155748.

[66] S. Han , Y. Hu , J. Wei , S. Li , P. Yang , H. Mi , C. Liu , C. Shen , Adv. Funct. Mater. 2024, 34, 2401607.

[67] J. Ren , G. Chen , H. Yang , J. Zheng , S. Li , C. Zhu , H. Yang , J. Fu , Adv. Mater. 2024, 36, 2412162. 10.1002/adma.202412162 39388508

[68] C. Zhao , X. Gong , L. Shen , Y. Wang , C. Zhang , ACS Appl. Polym. Mater. 2022, 4, 4025.

[69] X. Wang , H.‐J. Kim , Prog. Org. Coat. 2022, 166, 106784.

[70] Y. Xu , K. Sun , L. Huang , Y. Dai , X. Zhang , F. Xia , ACS Appl. Mater. Interfaces 2024, 16, 10556. 38359102 10.1021/acsami.3c19373

[71] L. Wang , W. Xia , Y. Yu , S. Liu , Y. Peng , Z. Wu , H. Chen , J. Mater. Chem. C 2023, 11, 6627.

[72] C. Zhou , Y. Yu , W. Xia , S. Liu , X. Song , Z. Wu , H. Chen , Soft Matter 2023, 19, 9460. 38018427 10.1039/d3sm01073b

[73] X. Y. Tao , K. H. Zhu , H. M. Chen , S. F. Ye , P. X. Cui , L. Y. Dou , J. Ma , C. Zhao , J. He , P. Z. Feng , Mater. Today Chem. 2023, 32, 101624.

[74] B. Huang , W. Liu , Y. Lan , Y. Huang , L. Fu , B. Lin , C. Xu , Chem. Eng. J. 2024, 480, 147888.

[75] Q. Zhang , Q. Wang , G. Wang , Z. Zhang , S. Xia , G. Gao , ACS Appl. Mater. Interfaces 2021, 13, 50411. 34647459 10.1021/acsami.1c15784

[76] L. Fan , J. Xie , Y. Zheng , D. Wei , D. Yao , J. Zhang , T. Zhang , ACS Appl. Mater. Interfaces 2020, 12, 22225. 32315157 10.1021/acsami.0c06091

[77] J. Yuan , Y. Zhang , G. Li , S. Liu , R. Zhu , Adv. Funct. Mater. 2022, 32, 2204878.

[78] W. Wang , Z. Ma , Z. Hu , Y. Long , F. Wu , X. Huang , F. U. Nisa , H. Liang , Y. Dong , J. Wang , M. Tahir , J. Xu , L. He , Adv. Funct. Mater. 2025, 2502844, 10.1002/adfm.202502844.

