# Supplementary material for: Chinese Nian Gao Inspired Textured Janus Hydrogel for Body Signal Sensing and Human Machine Interaction
Source: Adv Sci (Weinh). 2025 Jul 30;12(40):e09573. doi: 10.1002/advs.202509573 (PMC12561419; doi:10.1002/advs.202509573)
Supplement: Supplementary file 1 — Supporting Information [file ADVS-12-e09573-s003.docx]

**Supporting Information**

**Chinese Nian Gao Inspired Textured Janus Hydrogel for Body Signal Sensing and Human Machine Interaction**

*Haiyu Li^1^, Hui Zhang^1,3^*, Xinxin Liu^1^, Jing Jie^2^, Ming Yin^2^ and Jie Du^1^**

1 School of Materials Science and Engineering, Hainan University, Haikou 570228, China

2 State Key Laboratory of Digital Medical Engineering, Key Laboratory of Biomedical Engineering of Hainan Province, School of Biomedical Engineering, Hainan University, 572025, Sanya, Hainan, China

3 Shandong Key Laboratory of Preparation and Application of New Thermoplastic Elastomer Materials, Shandong, China

*Correspondence: huizhang5068@163.com (H. Zhang); dujie@hainanu.edu.cn (J. Du)

**Experimental Section**

*Materials*

Cellulose nanocrystals (CNC) were synthesized using the sulfuric acid method. Acrylic acid (AA, >99%) and polyvinylpyrrolidone (PVP) were obtained from Shanghai Macklin Biochemical Co., Ltd. (Shanghai, China). Ammonium persulfate (APS, ≥98%), aluminum chloride hexahydrate (AlCl_3_·6H_2_O, 97%) and glycerol (Gly, 99%) were provided by Aladdin Chemistry Co., Ltd. (Shanghai, China). All chemicals were used without any purification. The water used in all the experiments was purified via a Millipore purification apparatus.

*Preparation of hydrogels*

Initially, a suitable amount of AlCl_3_·6H_2_O and PVP were sequentially introduced into a preconfigured CNC-Gly binary solvent by stirring. Subsequently, AA monomers were integrated into the above mixture to obtain highly homogeneous and transparent hydrogel precursors. Finally, the precursors were polymerized into hydrogels at 60℃ for 6 h via one-pot free radical polymerization under the activation of a small quantity of APS. The prepared hydrogels with various mass ratios of CNC to Gly (5:0, 4:1, 3:2, 2:3, 1:4) were named as PPAlC_x_G_y_, where x and y represent the mass ratio of CNC and Gly, respectively. The detailed compositions of hydrogels have been listed in **Table S1**.

*Characterization*

Fourier transform infrared spectroscopy (FTIR, Frontier, PerkinElmer, USA) and X-ray photoelectron spectroscopy (XPS, Thermo Scientific K-Alpha, USA) with Al Kα source were employed to analyze the composition characteristic of hydrogels. Raman spectra were obtained from a laser scanning Raman microscope (In Via, USA) with the 785 nm laser over a wavelength range of 4000-100 cm^−1^, and an acquisition time of 10 s per accumulation. Scanning electron microscope (SEM, Verios G4 UC, USA) and transmission electron microscope (TEM, JEM-2100, Japan) were used to investigate the morphology. Crystal structure was examined using an X-ray diffractometer (XRD, Rigaku Smart Lab 9Kw, Japan). The rheological behaviors of PPAlC_x_G_y_ hydrogels were assessed on a rheometer (TA Discovery HR-2, USA) equipped with a parallel-plate geometry of 20 mm in oscillation mode at room temperature. The angular frequency (ω) was swept from 0.1 to 100 rad/s at a fixed strain of 1% and the energy storage modulus (G′) and the loss modulus (G") were recorded accordingly.

*Mechanical Test*

The mechanical tests of PPAlC_x_G_y_ hydrogels were performed on a universal testing machine (Gotech AI-7000-SU2, China) at a stretching speed of 100 mm/min at ambient temperature. The tested hydrogels were rectangular shape of 40 mm × 20 mm × 3 mm. All these tests were conducted for three times.

*Adhesion Test*

The adhesion strength of PPAlC_x_G_y_ hydrogels to various substrate materials was measured via the lap shear test by a universal testing machine (Gotech AI-7000-SU2, China) at room temperature. The dimension of the tested hydrogels were 40 mm × 20 mm × 3 mm. Initially, one piece of the substrate materials was stuck by two pieces of hydrogels with the bonding area of 20mm × 20 mm. Afterwards, the sample was pulled until the occurrence of separation. The experiment was carried out in triplicate.

*Self-healing Test*

Two PPAlC_x_G_y_ hydrogels were cut into halves and the two broken pieces of the different colored separated parts were put together to contact for a period of time. In addition, the hydrogels before and after self-healing were measured using a universal testing machine. The test was performed in triplicate.

*Electrical Test*

A digital source meter (Keithley DMM6500, Tektronix, USA) was used to record the electrical conductivity and the electrical signals of PPAlC_x_G_y_ hydrogels based on different strains. The electrical conductivity is estimated as follows Equation (1):

σ = L/AR (1)

where L, R, and A represent the thickness, resistance, and cross-sectional area of the hydrogels.

The data of electrical signals based on different strains were expressed in the form of the relative change of the resistance. The calculation of relative ratio of resistance changes was

conducted according to Equation (2):

ΔR/R_0_= (R - R_0_)/R_0_ (2)

where R_0_ and R are the resistance at the original length and the resistance in a certain motion state, respectively.

The sensitivity can be determined using Equation (3):

Gauge factor (GF)=ΔR/R_0_ε (3)

where ε was the strain during the test.

The results were averaged based on three independent measurements.

*Biocompatibility Test*

The MTT (3-(4,5-dimethylthiazol-2-yl)-2,5-diphenyltetrazolium bromide) tetrazolium assay was used to assess the biocompatibility of PPAlC_x_G_y_ hydrogel. In brief, the L929 cells were diluted to obtain a cell density of 1×10^4^ cells/mL and then inoculated at 96-well plates with 100 μL per well. The plates were then incubated with 5% CO_2_ at 37 ℃ for 24 h. During the incubation process, the experimental groups were hydrogel-containing media with various concentrations (50, 100, 200, 500, and 1000 μg/mL) and the control group was standard medium (0 μg/mL).

The optical density value at 570 nm was measured using a microplate reader to detect the cell viability. Cell viability was calculated by the following Equation (4):

Cell viability (%) = (A sample)/(A control) × 100% (4)

Each group was tested in triplicate. In addition, the laser confocal microscopy was employed to observe the morphology of L929 cells.

*Wearable sensing and human-machine interaction*

The sensing performance of the PPAlC_x_G_y_ hydrogel strain sensor was evaluated by measuring the resistance changes under various limb postures. In the integrated electrical system, a voltage applied to the series circuit is converted into driving signals for the motors of the assistive robotic arm based on the voltage measured across each sensor. Specifically, during operation, the closed-loop control system applies voltage to the series circuit. The voltage from the tactile sensors is converted into Direct Current (DC) values and transmitted to the processing unit of the control system. In the closed-loop control of the assistive robotic arm, the collected voltage serves as a feedback signal to the motor drivers, which in turn control the movements of the assistive robotic arm. A similar approach is used for controlling the assistive robotic arm with an attention assessment feedback control system based on EEG signals, where the predefined attention score threshold is set at 20.

Statistical Analysis

All experimental data are presented as mean values with error bars representing the standard deviation. Each measurement is conducted with n=3 independent samples, and the reported values reflect the average across these replicates. Given the limited sample size, statistical hypothesis testing is not performed, as the results may not be statistically robust.


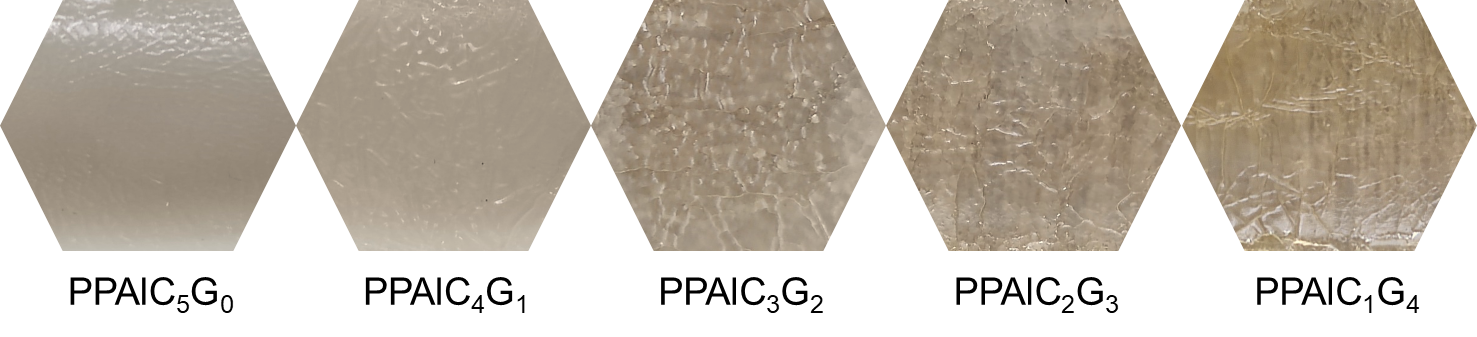


**Figure S1.** The tunable transparency and texture of the PPAlC_x_G_y_ hydrogel via the ratio of CNC to Gly.

**
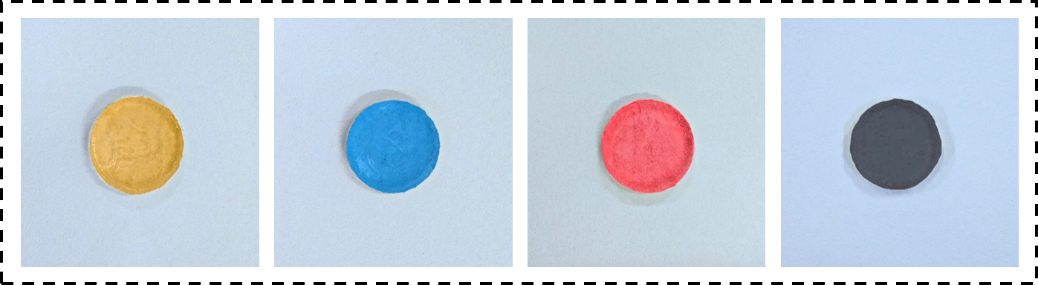
**

**Figure S2.** The various colors of PPAlC_x_G_y_ hydrogel after dyeing.





**Figure S3.** Raman spectra of PPAlC_x_G_y_ hydrogels.


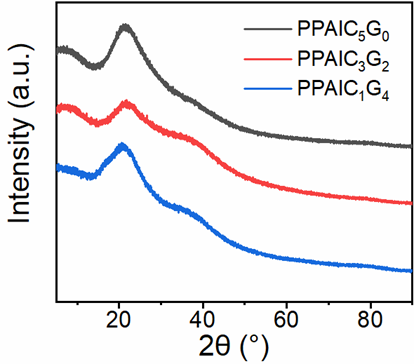


**Figure S4.** XRD patterns of PPAlC_x_G_y_ hydrogels.





**Figure S5.** Pore size statistic of PPAlC_x_G_y_ hydrogels.


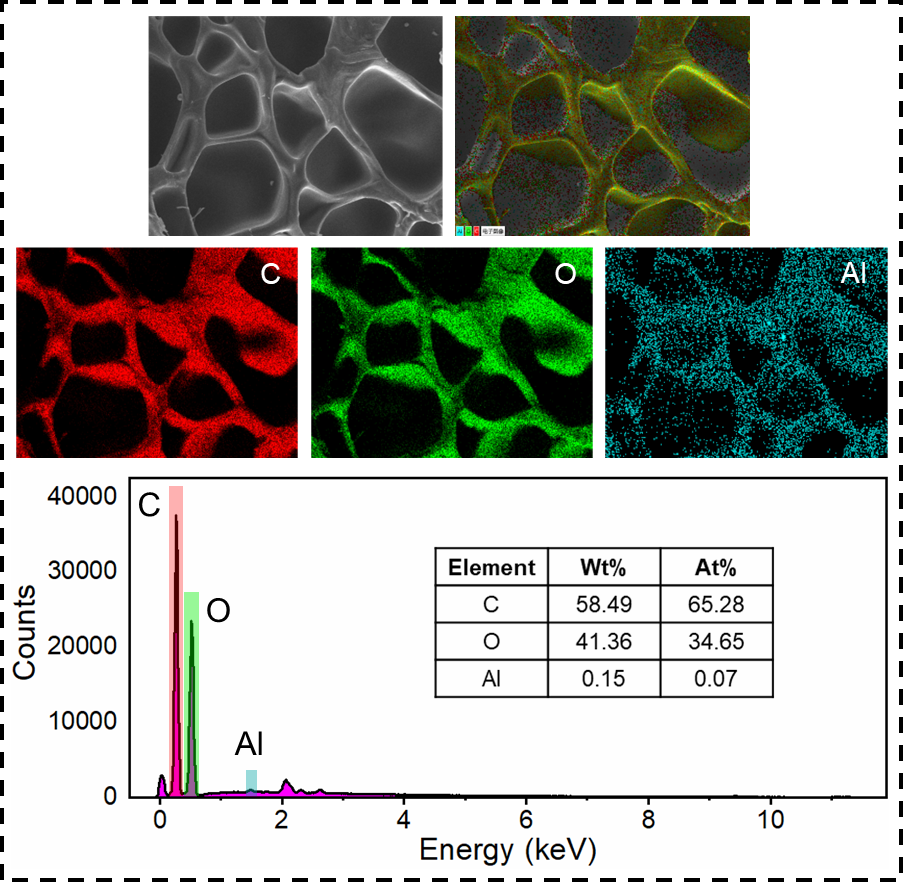


**Figure S6.** Element mapping of PPAlC_x_G_y_ hydrogels.


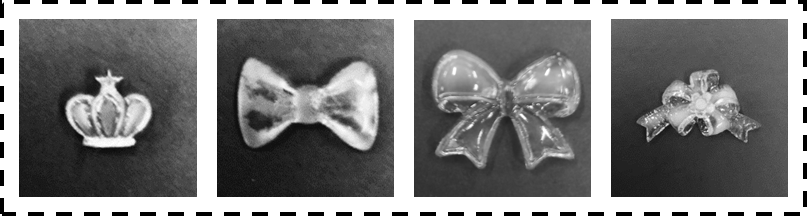


**Figure S7.** Optical photographs of PPAlC_x_G_y_ hydrogels in various shapes.





**Figure S8.** XPS survey spectra of the adhesive surface and non-adhesive surface of PPAlC_x_G_y_ hydrogels.


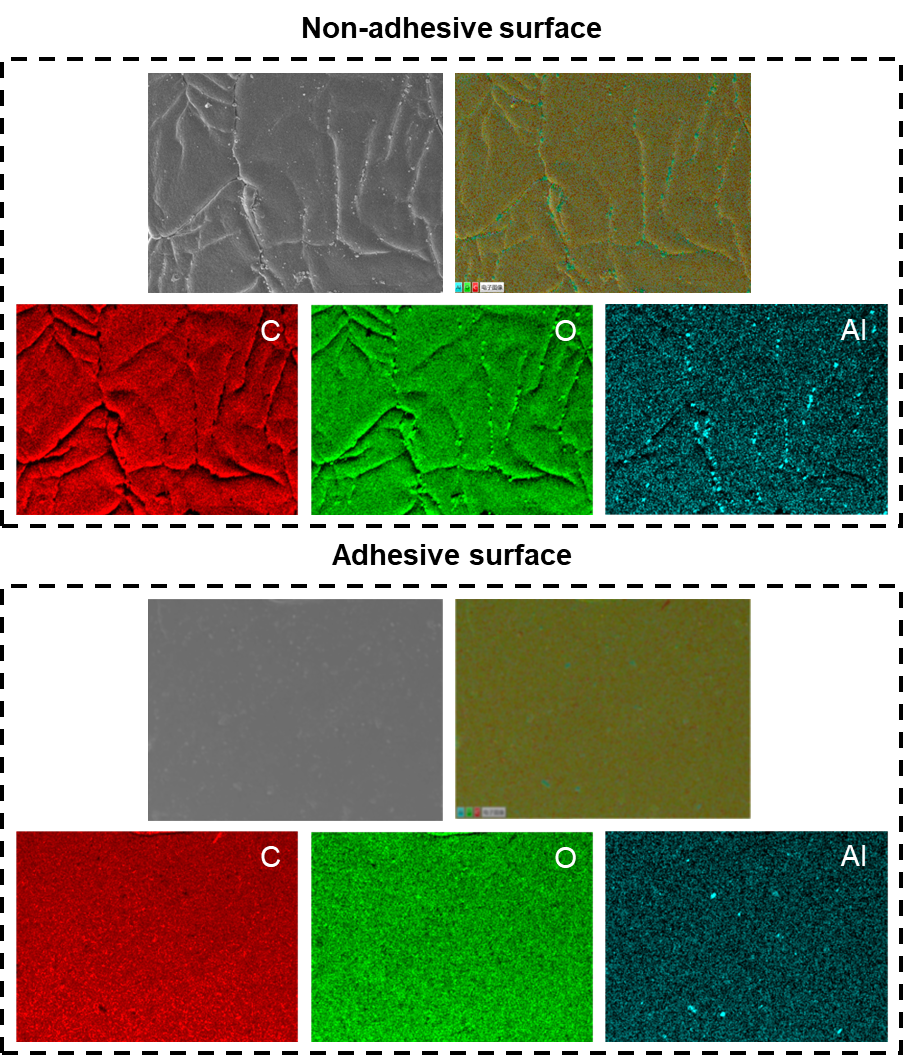


**Figure S9.** EDS analysis of non-adhesive surface and adhesive surface for PPAlC_x_G_y_ hydrogels.


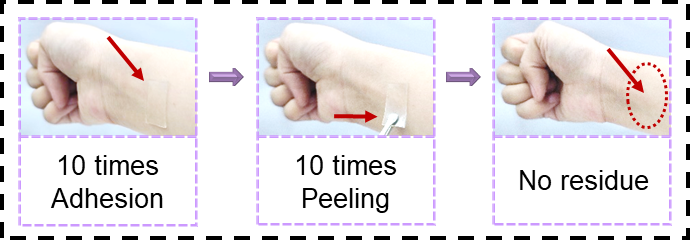


**Figure S10.** Adhesion and peeling tests for 10 times.


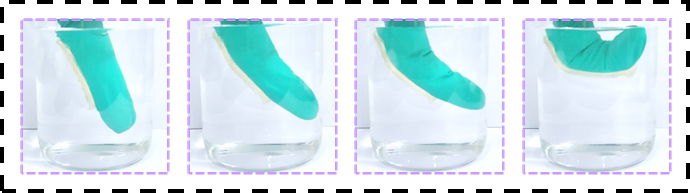


**Figure S11.** Underwater adhesive behavior of PPAlC_x_G_y_ hydrogel for sticking to the gloved fingers and bending without falling off.


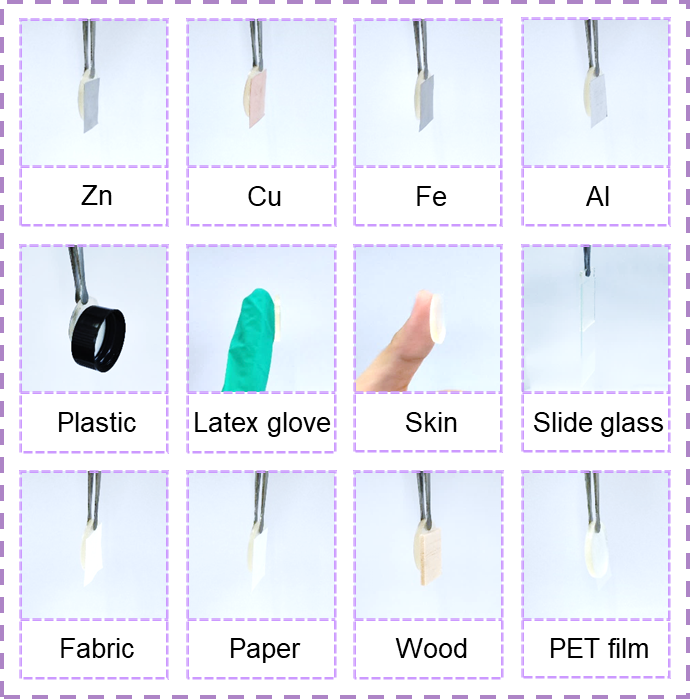


**Figure S12.** Photographs of the adhesive side of PPAlC_x_G_y_ hydrogels adhering on the surface of various substrates.


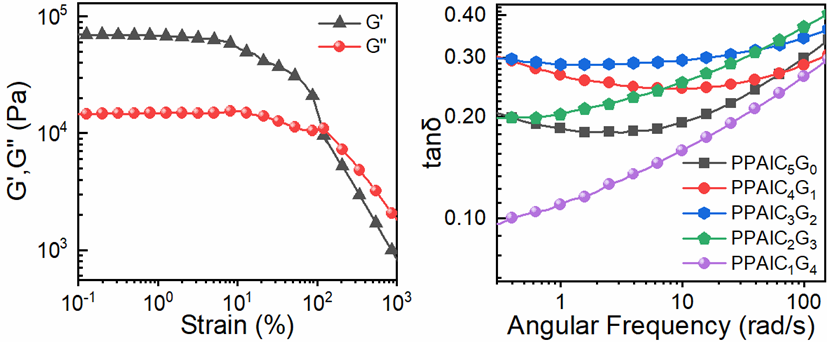


**Figure S13.** Dynamic viscoelasticity performance of PPAlC_x_G_y_ hydrogels. (a) Strain dependence of G' and G''. (b) Angular frequency dependence of tan δ.





**Figure S14.** Conductivity of PPAlC_x_G_y_ hydrogels.


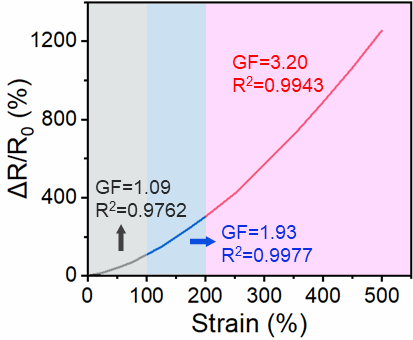


**Figure S15.** GF of the PPAlC_x_G_y_ hydrogels within the 500 % strain range.





**Figure S16.** Comprehensive performance comparison in sensitivity (GF), sensing range, response time, asymmetric Janus adhesion, self-healing capacity, textured structures and asymmetric adhesion between PPAlC_x_G_y_ hydrogels and representative hydrogel sensors.


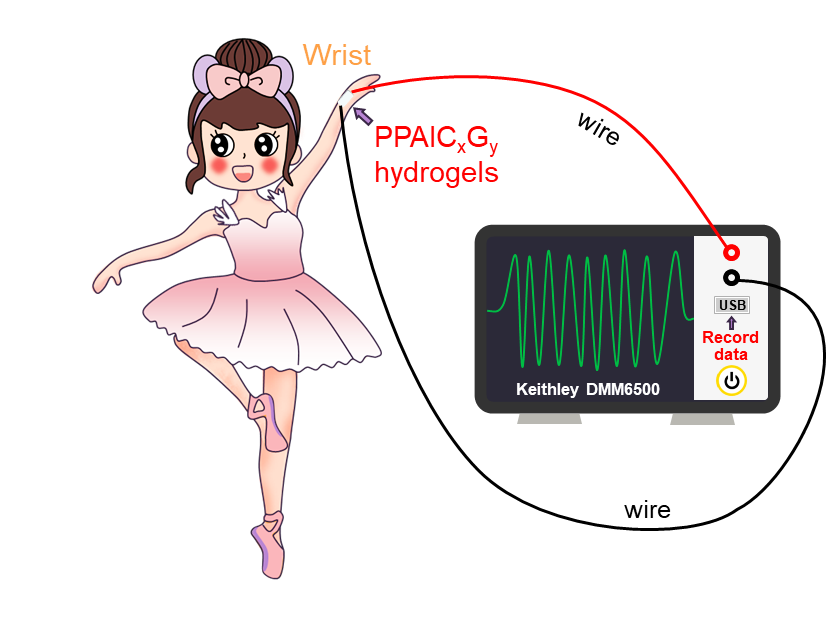


**Figure S17.** Schematic illustration of the experimental setup for real-time motion signals detection using the PPAlC_x_G_y_ hydrogel-based sensor.





**Figure S18.** The electromechanical output curves at 40%, 60%, and 80% relative humidity (RH) under room temperature for PPAlC_x_G_y_ hydrogels.


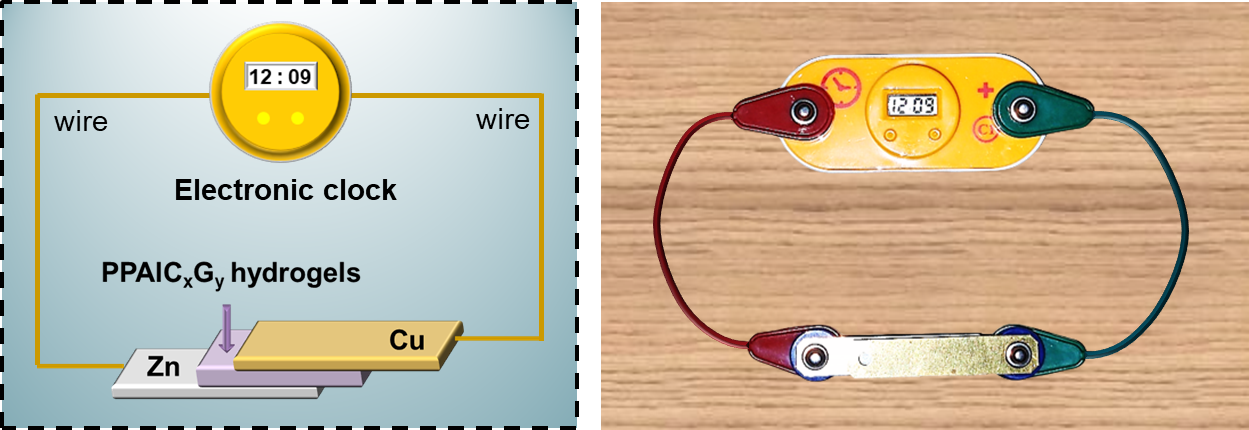


**Figure S19.** The sensor device for powering electronic clock. Successful current delivery to the electronic clock by the hydrogel-mediated battery circuit verifies the high conductivity of PPAlC_x_G_y_ hydrogel and its self-powering property. Here, the PPAlC_x_G_y_ hydrogel was sandwiched between a top copper sheet and a bottom zinc sheet, collectively forming a primary battery structure.

**Table S1.** The compositions of PPAlC_x_G_y_ hydrogels.

| Samples | CNC:Gly  (vol.%) | AlCl_3_·6H_2_O/AA  (mol.%) | PVP  (wt%) | APS  (wt%) |
| --- | --- | --- | --- | --- |
| PPAlC_5_G_0_ | 5:0 | 1.2 | 3 | 0.5 |
| PPAlC_4_G_1_ | 4:1 | 1.2 | 3 | 0.5 |
| PPAlC_3_G_2_ | 3:2 | 1.2 | 3 | 0.5 |
| PPAlC_2_G_3_ | 2:3 | 1.2 | 3 | 0.5 |
| PPAlC_1_G_4_ | 1:4 | 1.2 | 3 | 0.5 |

**Table S2.** Comprehensive performance comparison in sensitivity (GF), sensing range, response time, asymmetric Janus adhesion, self-healing capacity, textured structures and asymmetric adhesion between PPAlC_x_G_y_ hydrogels and representative hydrogel sensor.

| **Material** | **Sensing range**  **(%)** | **GF** | **Response time**  **(s)** | **Self-healing** | **Asymmetric Janus adhesion** | **Textured structures** | **Ref** |
| --- | --- | --- | --- | --- | --- | --- | --- |
| PVA/PSBMA-H_2_SO_4_ | 0-150  150-300 | 0.15  0.36 | 0.16 | - | - |  | ^[67]^ |
| CA-Ca^2+^ | 0-55 | 0.23 | - | - | - | - | ^[68]^ |
| PAAM/SA/LiCl/  CaCl_2_/glycerin | 0-100  100-300 | 0.45  2.31 | - | - | - | - | ^[69]^ |
| PAAm/Alg-Ca | 0-100  100-600 | 0.84  0.65 | - | - | Yes | - | ^[70]^ |
| PDMA-RSF | 0-100  100-300  300-500 | 0.65  1.39  2.24 | 0.40 | - | Yes | - | ^[71]^ |
| PDMA-Zr^4+^/[BMIM][Tf_2_N] | 0-100  100-200  200-300 | 1.85  2.45  2.75 | 0.36 | Yes | Yes | - | ^[72]^ |
| SF/PVA/Gly/LiCl | 0-200 | 2.18 | 0.20 | - | - | - | ^[73]^ |
| PVA-SA-FT | 0-195 | 2.01 | - | - | - | - | ^[74]^ |
| HF(PVA-C/P) | 0-400 | 2.10 | 0.20 | - | - | - | ^[75]^ |
| PVA/G/PDA/AgNPs | 0-70  70-315 | 0.94  0.13 | - | - | - | - | ^[76]^ |
| PPAlC_x_G_y_ | 0-100  100-200  200-500 | 1.09  1.93  3.20 | 0.13 | Yes | Yes | Yes | **This work** |

**Reference**

[67] J. Ren, G. Chen, H. Yang, J. Zheng, S. Li, C. Zhu, H. Yang, J. Fu, *Adv. Mater.* **2024**, *36*, 2412162.

[68] C. Zhao, X. Gong, L. Shen, Y. Wang, C. Zhang, *ACS Appl. Polym. Mater.* **2022**, *4*, 4025.

[69] X. Wang, H.-J. Kim, *Prog. Org. Coat.* **2022**, *166*, 106784.

[70] Y. Xu, K. Sun, L. Huang, Y. Dai, X. Zhang, F. Xia, *ACS Appl. Mater. Interfaces* **2024**, *16*, 10556.

[71] L. Wang, W. Xia, Y. Yu, S. Liu, Y. Peng, Z. Wu, H. Chen, *J. Mater. Chem. C* **2023**, *11*, 6627.

[72] C. Zhou, Y. Yu, W. Xia, S. Liu, X. Song, Z. Wu, H. Chen, *Soft Matter* **2023**, *19*, 9460.

[73] X. Y. Tao, K. H. Zhu, H. M. Chen, S. F. Ye, P. X. Cui, L. Y. Dou, J. Ma, C. Zhao, J. He, P. Z. Feng, *Mater. Today Chem.* **2023**, *32*, 101624.

[74] B. Huang, W. Liu, Y. Lan, Y. Huang, L. Fu, B. Lin, C. Xu, *Chem. Eng. J.* **2024**, *480*, 147888.

[75] Q. Zhang, Q. Wang, G. Wang, Z. Zhang, S. Xia, G. Gao, *ACS Appl. Mater. Interfaces* **2021**, *13*, 50411.

[76] L. Fan, J. Xie, Y. Zheng, D. Wei, D. Yao, J. Zhang, T. Zhang, *ACS Appl. Mater. Interfaces* **2020**, *12*, 22225
